# Sensory processing sensitivity and overstimulation in daily life: an experience sampling method study

**DOI:** 10.1038/s41598-025-31629-3

**Published:** 2025-12-18

**Authors:** Sofie Weyn, Corina U. Greven, Stefanie J. Schmidt, Céline R. Gillebert

**Affiliations:** 1 Division of Clinical Child and Adolescent Psychology, University of Bern, Fabrikstrasse 8, 3012 Bern, Switzerland; 2 Department of Brain & Cognition, Leuven Brain Institute, KU Leuven, Tiensestraat 102, 3000 Leuven, Belgium; 3 Radboud University Medical Center, Donders Institute for Brain, Cognition and Behaviour, Department of Medical Neuroscience, Nijmegen, Kapittelweg 29, 6525 EN Nijmegen, The Netherlands; 4 Karakter Child and Adolescent Psychiatry University Centre, Reinier Postlaan 12, 6525 GC Nijmegen, The Netherlands; 5 King’s College London, Institute of Psychology, Psychiatry and Neuroscience, Social, Genetic and Developmental Psychiatry, DeCrespigny Park, SE5 8AF London, United Kingdom

**Keywords:** Sensory processing sensitivity, Overstimulation, Experience sampling methodology, Triggers, Fluctuations, Health care, Neuroscience, Psychology, Psychology

## Abstract

Individuals differ in how they perceive and process social and sensory information in their environment, a personality trait known as *Sensory Processing Sensitivity* (SPS). Approximately 30% of the general population scores high on this trait making them more responsive to both negative and positive environmental influences than individuals lower in SPS. *Overstimulation* is one of the biggest challenges associated with SPS. Associations between SPS, triggers, and fluctuations of overstimulation in everyday life were examined using an experience sampling method study (during one week with 5 prompts a day) in 139 healthy adults. Results showed that overstimulation increased in the afternoon to early evening and in the presence of others. Furthermore, more sensitive individuals reported *higher* levels of overstimulation when auditory and visual stimuli were rated as unpleasant, when fatigued, or in a negative mood. Yet, more sensitive individuals reported *lower* levels of overstimulation with momentary pleasant auditory and visual stimuli, when not fatigued, and in a positive mood at the current moment. Everyone, but particularly individuals high in SPS, may benefit from interventions preventing fatigue, promoting positive mood and the pleasantness of sensory stimuli in their daily environments to reduce feelings of overstimulation.

**Supplementary Information:**

The online version contains supplementary material available at 10.1038/s41598-025-31629-3.

## Introduction

Approximately 30% of the general population scores high on the personality trait Sensory Processing Sensitivity (SPS), also referred to as Highly Sensitive Persons (HSPs)^1^. SPS is characterized by lower sensory thresholds, susceptibility to overstimulation, deeper processing of environmental information, heightened emotional and physiological reactivity, and increased awareness of subtle stimuli^2,3^. Genetic and neuroimaging studies indicate that differences in SPS are moderately heritable (47%) and driven by a more sensitive nervous system^[e.g.,2,5–7^. While distinct from the Big Five personality traits, SPS is associated with *openness* and *neuroticism*^8,9^. SPS is a dimensional trait^10,11^, with research suggesting that 20–30% of the individuals fall into a highly sensitive, 40–50% into a medium sensitive, and 20–30% into a low sensitive group^10,12^. Individuals higher on SPS are more affected by negative and positive environments than individuals lower on SPS^1,3^. Evidence showed worse outcomes (e.g., a lower quality of life, physical health complaints, and burnout) in adverse environments^13–15^, but better outcomes in supportive environments (e.g., psychological interventions and positive affect)^10,13,16,17^. This dual sensitivity algins with the differential susceptibility model^18,19^, incorporating both Diathesis Stress (‘for worse’)^20^ and Vantage Sensitivity (‘for better’)^17,21^. Evidence supports the coexistence of these models, with findings varying by the studied population, the sensitivity marker, environmental context, outcomes, and methodology^3,22–24^.

### Overstimulation in the general population

Overstimulation or sensory overload refers to excessive or atypical stimulation that exceeds an individuals’ usual thresholds^25^. It can arise from both objective (e.g., noise produced by large crowds) and subjective (e.g., someone’s expectations about a current situation) factors^25,26^. When sensory input (e.g., sounds) surpasses one’s capacity to process it, whether due to volume, intensity or diversity of stimuli, stimuli become aversive and can result in overstimulation^25,27^. Overstimulation can result from stimulation in one or multiple sensory modalities^25,26,28,29^, which can be categorized into high (i.e., vision and audition) and low senses (i.e., smell, taste and touch)^30^. Research shows that primarily high sense stimuli with a high intensity (e.g., bright lights) compared to low sense stimuli contribute to overstimulation^30,31^. An individual’s susceptibility to overstimulation also depends on their psychobiological resources, shaped by genetics, personality traits (e.g., SPS) and the environmental context^25,26,28^. Personal states like fatigue^26,32^ and negative mood can increase an individual’s proneness to overstimulation, as they hinder the suppression of irrelevant information^26^. Overstimulation has been mostly examined in relation to clinical populations such, such as Autism Spectrum Disorder (ASD)^33^, Attention Deficit Hyperactivity Disorder (ADHD)^34^, and in patients with acquired brain injury^26^. Yet, also in non-clinical populations overstimulation can reach comparable severity levels^25,28,33^. In both populations, overstimulation may negatively impact physical (e.g., immunity), mental (e.g., depression and anxiety), cognitive (e.g., decision making), and social (e.g., isolation) wellbeing^13,25,27,31,35^. Despite its impact, overstimulation in non-clinical groups is under-researched. In today’s sensory-rich environments (e.g., new technologies, civilization), understanding its triggers and the psychobiological resources to adequately deal with the demands of sensory environments is increasingly important^25,27,28^.

### Overstimulation in relation to sensory processing sensitivity

Overstimulation is one of the most frequently reported challenge among individuals high on SPS^36,37^. Due to lower sensory thresholds, heightened emotional reactivity, and deeper processing, individuals higher on SPS may have lower psychobiological resources and respond more strongly to the same sensory input (e.g., background noise, artificial lights, other’s emotions) than individuals lower on SPS^1,3^. This means that even low levels of sensory input (e.g., background noise) can lead to overstimulation in individuals higher on SPS^2,38^. The SPS framework suggests that mainly negatively perceived stimulation can trigger overstimulation, whereas positively perceived stimulation (e.g., music) can enhance wellbeing^3,38^. In a qualitative study^36^, individuals high on SPS reported strong emotional and cognitive responses to both positive and negative situations, sensitivity to others’ emotions, attention to detail, and frequent fatigue and stress. Feelings of overstimulation may fluctuate more in individuals higher on SPS than in less sensitive ones, depending on their exposure to daily positive (e.g., pleasant scents) and negative environments (e.g., background noise in large offices). Retrospective studies partially supported this idea. Iimura^39^ found that individuals higher on SPS reported greater fluctuations in well-being linked to weekly positive and negative events. However, Damatac^14^ found that SPS was related to more daily negative but not to more daily positive events and Van Reyn^40^ found that individuals higher on SPS showed stronger reactions to daily negative but not to positive events. The latter^40^ was the first study examining the association between SPS and the reactivity to daily positive and negative events by using a daily diary study. In the above studies, positive and negative events were measured retrospectively only once^14^, once per day^40^, or once per week^39^. In contrast, the present study examines reactivity, in terms of stimulation, to momentary environments and personal states in daily life using Experience Sampling Methodology (ESM)^41,42^. ESM is a structured moment-to-moment diary method in which individuals respond to brief questions about their current environment (i.e., external triggers) and momentary personal states (i.e., internal triggers) multiple times a day during a specific time frame. This approach minimizes the susceptibility to negativity and recall biases (i.e., the tendency to remember negative events more than positive ones), which are common in traditional questionnaires and retrospective studies^42,43^.

## The present study

The aim of the current study was to examine triggers of and fluctuations in overstimulation in healthy individuals high on SPS (hereafter referred to as Highly Sensitive Persons (*HSPs*)) compared to individuals low or average on SPS (hereafter referred to as *non-HSPs*). Ease of overstimulation is conceptualized as a key characteristic of SPS^1^. However, the current study was the first to empirically examine this relation in greater depth using ESM. The first objective of the present study was to examine whether levels of overstimulation fluctuate more throughout the day and the week in HSPs compared to non-HSPs. We expected higher fluctuations in overstimulation depending on the environments HSPs encounter. The second objective was to explore which positive and negative external and internal triggers are associated with overstimulation in HSPs compared to non-HSPs. Identifying these triggers may provide insights into the underlying mechanisms of overstimulation and thereby on the development of evidence-based interventions targeted to a specific group of individuals high on SPS. Based on key characteristics of SPS, we expected that HSPs, compared to non-HSPs, would be more easily overstimulated in response to negatively perceived stimulation, but less overstimulated in response to positively perceived stimuli. However, due to the lack of evidence, we did not have specific hypotheses regarding particular triggers of overstimulation.

## Results

### Preliminary and descriptive analyses

The within-person and between-person means, standard deviations, the response rates per variable, and Pearson correlations are reported in Tables A1 and B1 (Supplementary Material A and B). The mean response rate was 86.20% (*SD* = 16.50) and did not significantly differ (Wilcoxon rank–sum test = 2594, *p* = .44) between the HSP (84.88%; *SD* = 17.18) and non-HSP group (87.56%; *SD* = 15.69).

### Fluctuations in overstimulation during the day and the week

Overstimulation showed a non-linear pattern across the day. The significant positive linear and negative quadratic effects indicate an inverted-U trajectory with increasing levels from morning to early afternoon (prompts 1 and 2), with a peak between 1 and 7pm (prompts 3 and 4), and decreasing levels in the evening (between prompts 4 and 5; Fig. 1). SPS did not significantly moderate this pattern (Table 1), meaning that HSPs and non-HSPs displayed similar daily dynamics, although there was a non-significant trend for HSPs to report higher overstimulation across all time intervals (Fig. 1). Weekday effects were not significant (Table 1). The fluctuations of overstimulation during the day nested in weekday are visualized in Fig. 2.

**Figure:**
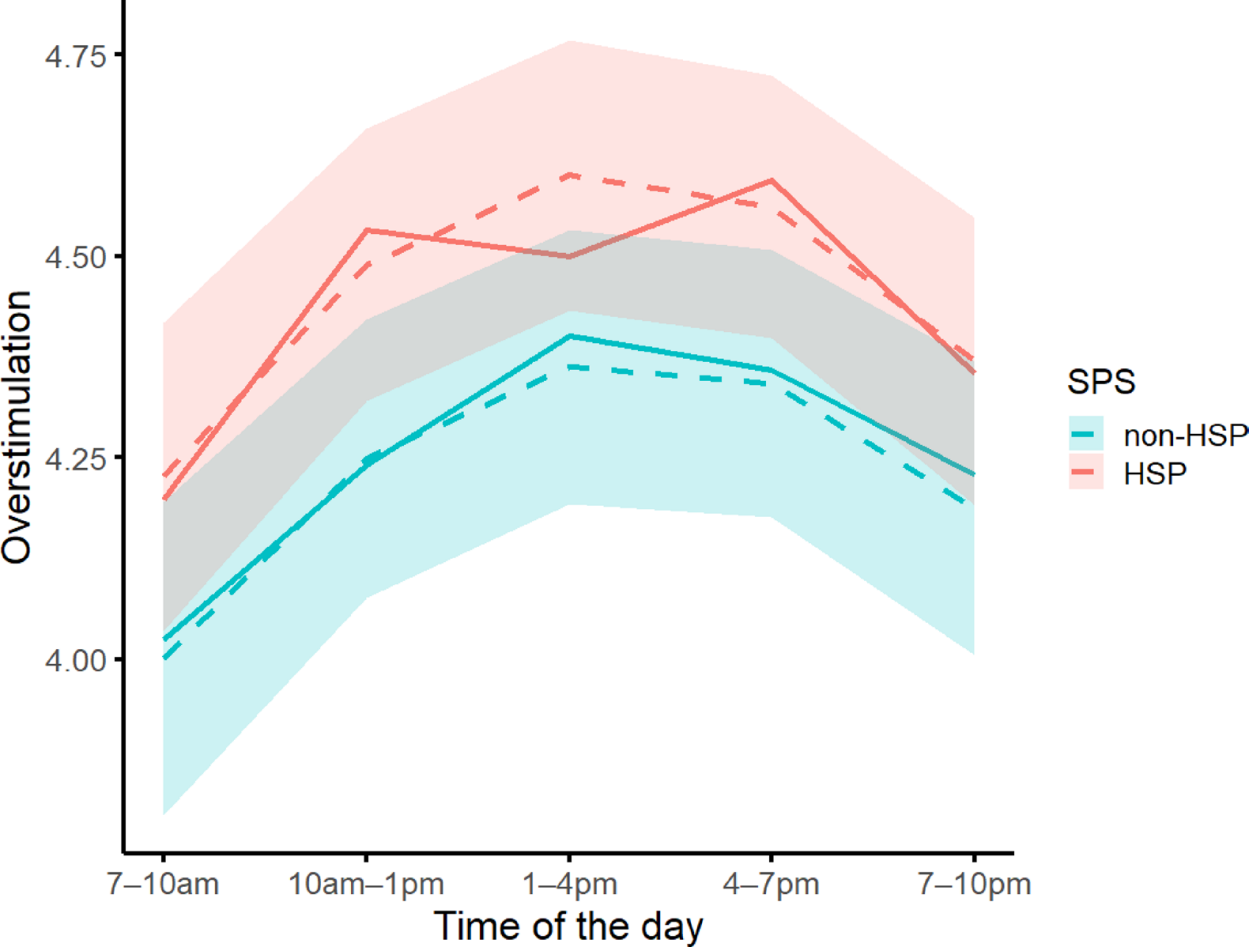
**Fig. 1** Fluctuations in Overstimulation During the Day. The full lines represent the raw data and the dashed lines represent the estimated (i.e., visualization of the results in Table 1) data. The estimated confidence intervals are plotted around the estimated lines. The X-axis represents the time intervals in which the prompts were sent, averaged over the days of the week. These time-intervals were semi-randomized within two-hour blocks between 7am and 9pm or between 8am and 10pm, based on participants’ self-identified morning or evening rhythms.

**Table 1:** Multilevel random intercept and slope model results predicting fluctuations in overstimulation (Model 1).

Model 1	Β (SE)	*p* -value
Intercept	4.07 (0.10) 95% CI [3.87; 4.07]	< 0.001
Time of the day	0.45 (0.08) 95% CI [0.29; 0.45]	< 0.001
Time of the day²	-0.07 (0.01) 95% CI [-0.09; -0.07]	< 0.001
Weekday	-0.07 (0.05) 95% CI [-0.03; 0.03]	0.17
SPS	0.23 (0.14) 95% CI [-0.03; 0.50]	0.09
Time of the day*SPS	0.04 (0.12) 95% CI [-0.19; 0.27]	0.72
Time of the day²*SPS	-0.01 (0.02) 95% CI [-0.05; 0.03]	0.64
Weekday *SPS	-0.01 (0.08) 95% CI [-0.05;0.03]	0.78
ICC	0.22	
*R*² (fixed effects)	0.02	
*R*² (total)	0.23	

*SPS* Sensory Processing Sensitivity, categorized as HSP versus non-HSP; *Time of the day* refers to the different prompts (1 to 5) within a day.* Time of the day²* is the quadratic term of time of the day. *ICC* Intraclass correlation coefficient. *R²* proportion of variance explained by the fixed effects and/or fixed and random effects. *CI* confidence interval. *p*-values are two-sided.

**Figure:**
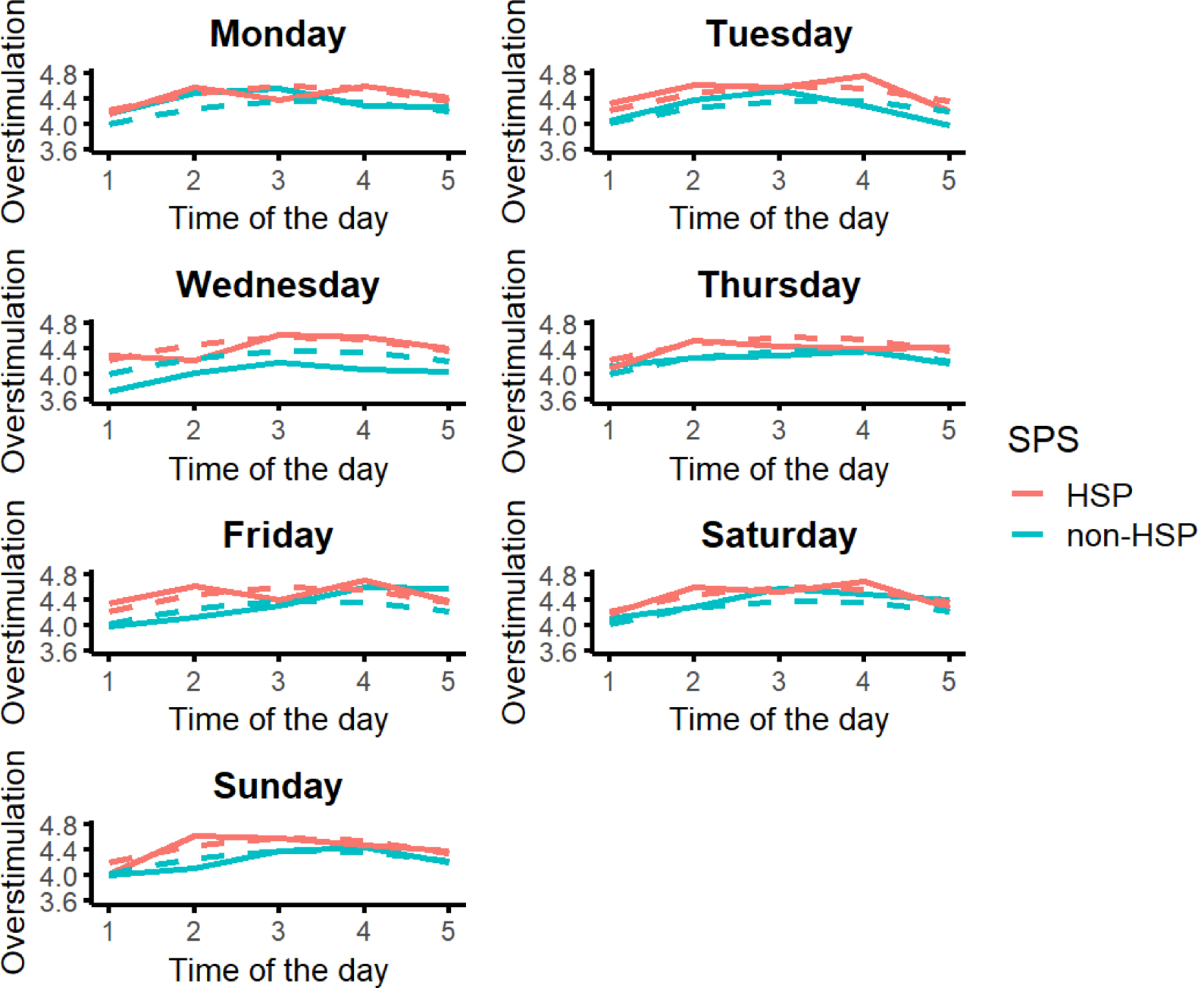
**Fig. 2.** Fluctuations in Overstimulation During the Day Across the Different Days of the Week. The full lines represent the raw data and the dashed lines represent the estimated (i.e., visualization of the results in Table 1) data. The X-axis represents the time intervals in which the prompts were sent. These were semi-randomized within two-hour blocks between 7am and 9pm or between 8am and 10pm, based on participants’ self-identified morning or evening rhythms: 1 = between 7 and 10am, 2 = between 10am and 1pm, 3 = between 1 and 4pm, 4 = between 4 and 7pm, 5 = between 7-10pm.

### The prediction of overstimulation by sensory processing sensitivity, the pleasantness of stimuli in the current environment, the type of environment, mood, fatigue, and its interactions

**Fig. 3:**
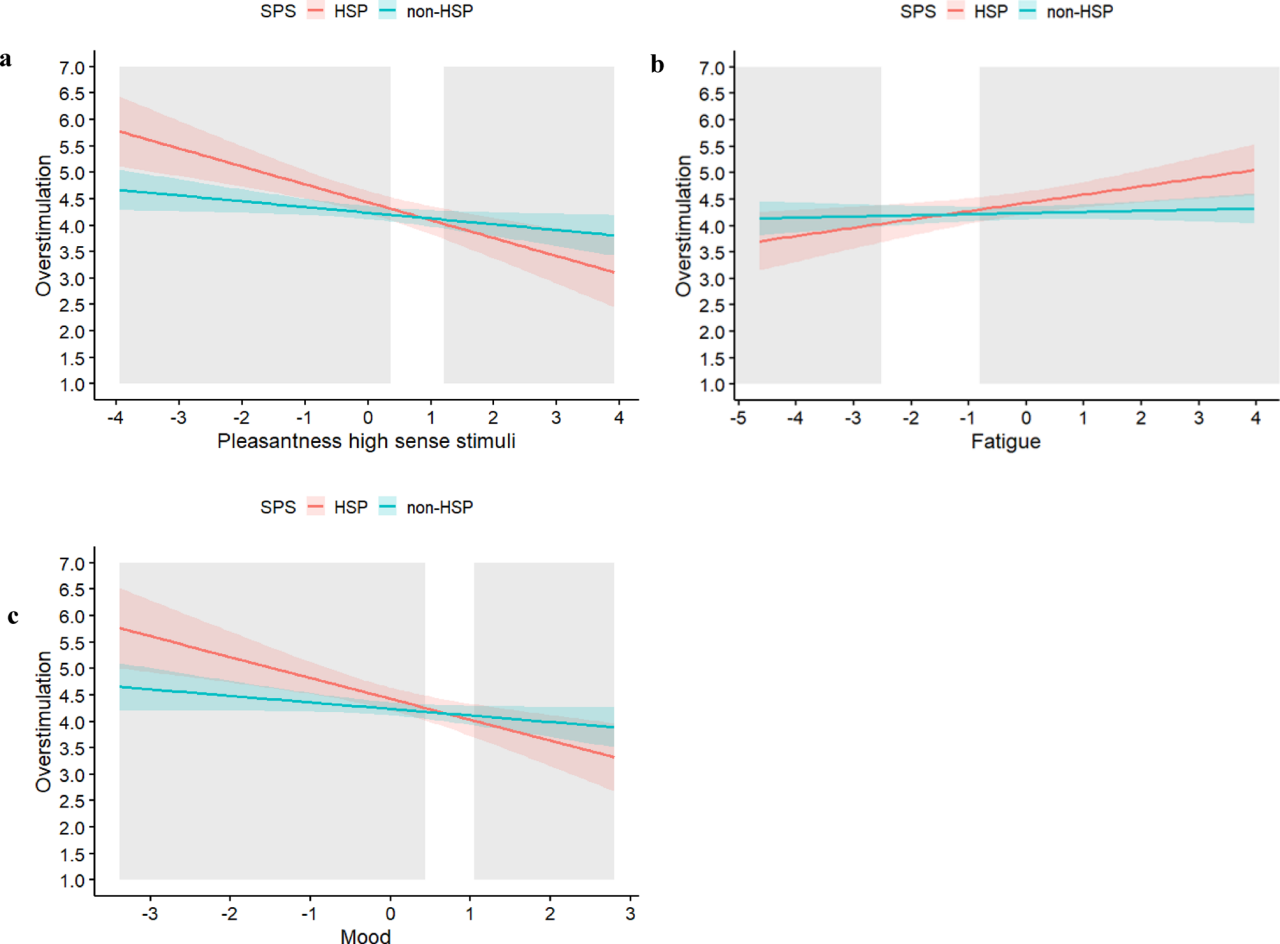
Overstimulation as Predicted by the Interactions of SPS and Pleasantness of High Sense Stimuli (a), Fatigue (b), and Mood (c) as Reported Momentarily. The grey areas represent the Regions of Significance with respect to X (i.e., Pleasantness high sense stimuli, Fatigue, or Mood). The predictors (X-axis) are within-person mean centered. Only the observed range of values with respect to X were plotted. The estimated confidence intervals are plotted around the estimated lines.

Regarding momentary observations, overstimulation was higher in HSPs than in non-HSPs, in the presence of unpleasant high and low sense stimuli (Table 2), in the presence of others (Table 3), and when fatigue levels were higher (Table 4). Regarding the interactions with SPS (Fig. 3), we found that HSPs were more affected (steeper slopes) by the pleasantness of high sense stimuli (Table 3), fatigue, and mood (Table 4) than non-HSPs. HSPs showed *higher* levels of overstimulation than non-HSPs when they rated the pleasantness of high sense stimuli as low, fatigue as high, and mood as negative. In contrast, HSPs showed *lower* levels of overstimulation than non-HSPs when they rated the pleasantness of high sense stimuli as high, fatigue as low, and mood as positive. The proportion of interaction (PoI) of these models indicated that 29, 28 and 21% (pleasantness high sense stimuli, fatigue, and mood, respectively) of the interaction occurred at the ‘for better’ side and the remaining percentages (i.e., 71, 72, and 79% respectively) at the ‘for worse’ side. Regarding the examined associations with lagged predictors at the previous prompt, results were similar but weaker (Tables 2, 3 and 4; Supplementary Material C, Fig. C1). Looking at interactions between the lagged predictors and SPS, HSPs showed *higher* levels of overstimulation than non-HSPs when rating the pleasantness of high sense stimuli at the previous prompt as low (Table 2) and when reporting a negative mood and high levels of fatigue at the previous prompt (Table 4). As an exploratory analysis, we controlled for age and sex differences. Across all models results remained similar when including main effects of age and sex (Supplementary Material D, Table D1-2).

**Table 2:** Multilevel random intercept and slope model results predicting overstimulation by SPS, the pleasantness of stimuli in the environment, and its interactions, including momentary and lagged variables (Model 2).

	Within-person centered predictors (momentary observations)		Time-lagged predictors (previous observation)	
	Β (SE)	*p* -value	Β (SE)	*p* -value
Intercept	4.24 (0.06) 95% CI [4.12; 4.36]	< 0.001	4.09 (0.20) 95% CI [3.69;4.49]	< 0.001
Pleasantness high senses	-0.11 (0.05) 95% CI [-0.20; -0.02]	0.021	0.04 (0.03) 95% CI [-0.03;0.10]	0.232
Pleasantness low senses	-0.10 (0.04) 95% CI [-0.17; -0.03]	0.006	0.01 (0.04) 95% CI [-0.07;0.08]	0.873
SPS	0.20 (0.09) 95% CI [0.03; 0.37]	0.025	0.90 (0.27) 95% CI [0.35;1.44]	0.001
Pleasantness high senses * SPS	-0.23 (0.07) 95% CI [-0.36; -0.11]	< 0.001	-0.16 (0.05) 95% CI [-0.26;-0.07]	0.001
Pleasantness low senses * SPS	0.02 (0.05) 95% CI [-0.08; 0.02]	0.679	-0.01 (0.06) 95% CI [-0.12;0.10]	0.882
ICC	0.25		0.12	
*R*² (fixed effects)	0.06		0.02	
*R*² (total)	0.30		0.21	

*SPS* Sensory Processing Sensitivity, categorized as HSP versus non-HSP; *ICC* Intraclass correlation coefficient; *R²* proportion of variance explained by the fixed effects and/or fixed and random effects; *CI* confidence interval; *p* -values are two-sided.

**Table 3:** Multilevel random intercept and slope model results predicting overstimulation by SPS, the type of environment, and its interactions, including momentary and lagged variables (Model 3).

	Within-person centered predictors (momentary observations)		Time-lagged predictors (previous observation)	
	Β (SE)	*p* -value	Β (SE)	*p* -value
Intercept	3.89 (0.07) 95% CI [3.69;4.23]	< 0.001	4.03 (0.14) 95% CI [3.74;4.32]	< 0.001
Alone versus with others	0.43 (0.14) 95% CI [0.15;0.70]	0.002	0.15(0.07) 95% CI [0.01;0.30]	0.021
Private versus public	0.42 (0.20) 95% CI [0.04;0.82]	0.030^	0.04 (0.07) 95% CI [-0.09;0.19]	0.495
SPS	0.21 (0.09) 95% CI [0.04;0.38]	0.017	0.13 (0.21) 95% CI [-0.28;0.54]	0.530
Alone versus with others * SPS	-0.17 (0.09) 95% CI [-0.35;0.01]	0.060	-0.11 (0.10) 95% CI [-0.33;0.11]	0.230
Private versus public * SPS	0.04 (0.13) 95% CI [-0.21;0.30]	0.730	0.14 (0.11) 95% CI [-0.07;0.35]	0.163
ICC	0.23		0.39	
*R*² (fixed effects)	0.06		0.01	
*R*² (total)	0.27		0.39	

*SPS* Sensory Processing Sensitivity, categorized as HSP versus non-HSP; Alone was coded as 0 and with others as 1; Private was coded as 0 and in public places (including the work or school environment) as 1; *ICC* Intraclass correlation coefficient; *R²* proportion of variance explained by the fixed effects and/or fixed and random effects; *CI* confidence interval; ^*p* was not significant anymore after controlling for multiple testing; *p* -values are two-sided.

**Table 4:** Multilevel random intercept and slope model results predicting overstimulation by SPS, fatigue, mood, and the interactions between predictors and SPS, including momentary and lagged variables (Model 4 and 5).

	Within-person centered predictors (momentary observations)		Time-lagged predictors (previous observation)	
	Β (SE)	*p* -value	Β (SE)	*p* -value
Fatigue (Model 4)				
Intercept	4.24 (0.06) 95% CI [4.11; 4.36]	< 0.001	4.51 (0.10) 95% CI [4.31;4.72]	< 0.001
Fatigue	0.02 (0.03) 95% CI [-0.04;0.08]	0.508	-0.06 (0.03) 95% CI [-0.11;-0.01]	0.024
SPS	0.19 (0.08) 95% CI [0.02;0.37]	0.031^	-0.09 (0.16) 95% CI [-0.40;0.22]	0.550
Fatigue *SPS	0.14 (0.05) 95% CI [0.05;0.22]	0.003	0.08 (0.04) 95% CI [0.01;0.15]	0.030
ICC	0.23		0.20	
* R*² (fixed effects)	0.02		0.01	
* R*² (total)	0.25		0.21	
Mood (Model 5)				
Intercept	4.24 (0.06) 95% CI [4.11;4.36]	< 0.001	3.98 (0.26) 95% CI [3.46;4.49]	< 0.001
Mood	-0.12 (0.07) 95% CI [-0.25;0.00]	0.058	0.06 (0.04) 95% CI [-0.03;0.15]	0.206
SPS	0.19 (0.08) 95% CI [0.02;0.37]	0.03^	1.02 (0.35) 95% CI [0.32;1.69]	0.004
Mood*SPS	-0.27 (0.09) 95% CI [-0.44;-0.10]	0.002	-0.16 (0.06) 95% CI [-0.28;-0.04]	0.009
ICC	0.26		0.21	
* R*² (fixed effects)	0.05		0.02	
* R*² (total)	0.30		0.22	

*SPS* Sensory Processing Sensitivity, categorized as HSP versus non-HSP; *ICC* Intraclass correlation coefficient; *R²* proportion of variance explained by the fixed effects and/or fixed and random effects; *CI* confidence interval; ^*p* was not significant anymore after controlling for multiple testing; *p* -values are two-sided.

## Discussion

The present study examined fluctuations in overstimulation and its triggers in healthy adults with varying levels on the personality trait SPS. Overstimulation is a frequently reported challenge associated with SPS but was not yet empirically studied in depth. The first objective was to examine whether feelings of overstimulation fluctuate more in individuals with high levels of SPS (HSPs) than in individuals with low or average levels of SPS (non-HSPs) throughout the day and week. The second objective was to identify daily external and internal triggers of overstimulation in both groups.

Regarding the first objective, results showed that levels of overstimulation fluctuated throughout the day, but not the week. Overstimulation showed a nonlinear pattern across the day, with increasing levels from the morning to early afternoon, a peak between 1 and 7pm, and a decrease toward the evening. The highest levels of overstimulation were reported in the afternoon (coinciding with the second half and the end of the workday) and then decreased again in the evening (likely as participants began winding down before bedtime). Contrary to our hypothesis, HSPs did not show greater fluctuations in overstimulation. Possible explanations include: (1) five daily ESM measures may not capture subtle changes, (2) HSPs may have reached ceiling levels, as seen by their higher average levels of overstimulation, and (3) HSPs may have developed strategies to minimize fluctuations in overstimulation, such as avoiding stimulus rich environments or maintaining a structured daily routine. Our results showed that HSPs spent more time in private than in public environments compared to non-HSPs, which may be a strategy to avoid stimulation.

Regarding the second objective, overstimulation was higher for both HSPs and non-HSPs in the presence of others, in the presence of unpleasant high and low sense stimuli, when fatigued, and when reporting a negative mood. Notably, overstimulation did not increase in public places. When examining differences between HSPs and non-HSPs, we found a cross-over effect. HSPs reported *higher* levels of overstimulation than non-HSPs when they reported higher pleasantness of high sense stimuli (visuals and sounds), high levels of fatigue, and a negative mood. Furthermore, HSPs reported *lower* levels of overstimulation than non-HSPs when they rated high sense stimuli as pleasant and when they reported low levels of fatigue and a positive mood. Non-HSPs’ levels of overstimulation were less affected by external and internal triggers. Additionally, we examined associations between overstimulation and triggers reported at the previous prompt (between 1 and 4 hours earlier, depending on the timing of prompts). Results remained similar but were weaker. This could be related to methodological constraints leading to a substantial reduction in the number of observations for lagged analyses compared to momentary analyses. Specifically, the first observation of the day could not be lagged to the last observation of the previous day, and any missing observations at the previous prompt led to additional missing data. Our findings built further on a previous daily diary study^40^, showing that HSPs react more to daily negative, but not positive events. However, our momentary approach likely captured positive experiences better than retrospective studies, which are often influenced by recall biases toward negative events and memory-experience gaps^44^.

In summary, HSPs experienced more overstimulation from negative triggers but also greater relief from momentary positive factors compared to non-HSPs. These results align with previous studies suggesting that more sensitive individuals are more affected by both negative and positive experiences, often described as a ‘for better and for worse’ pattern^19^. The ‘for better’ effects were present in responses to momentary pleasant high sense stimuli, momentary low levels of fatigue, and momentary positive mood. In contrast, the ‘for worse’ effects appeared particularly in relation to high levels of fatigue, unpleasant high sense stimuli, and negative mood. Across models, the proportion of interaction on the ‘for worse’ side was higher than the proportion of interaction on the ‘for better’ side. This ‘for-better-and for-worse’ sensitivity is in line with the broader Environmental Sensitivity framework^45^ that integrates Differential Susceptibility^19^, Biological Sensitivity to Context^46^, and Sensory Processing Sensitivity (SPS)^1^. All three theories converge on the idea that a minority of individuals (approximately 30%) are more responsive to environmental influences in a positive and negative way. In the present study, we focused on SPS because it is conceptualized as a behavioral phenotype of the broader environmental sensitivity framework and operationalized through observable or self-reported sensory processing differences (e.g., Sensory Processing Sensitivity Questionnaire^47^. The Differential Susceptibility Theory emphasizes heightened developmental plasticity and Biological Sensitivity to Context highlights individual differences in physiological reactivity (e.g., cortisol). The pattern observed in the present study, namely stronger negative reactivity alongside enhanced benefits from positive momentary contexts, is consistent with the current conceptual extension of the Differential Susceptibility model, integrating diathesis-stress (‘for worse’) and vantage sensitivity (‘for better’) processes^18,21,48^. Our results therefore provide daily life evidence for this dual sensitivity within the SPS phenotype.

The results of the current study have important implications for reducing feelings of overstimulation in daily life. Raising awareness (e.g., through psychoeducation) about daily fluctuations and triggers of overstimulation may improve predictability and control, reducing the mismatch between environmental demands and personal resources, and thus lowering overstimulation^26,28^. For example, recognizing patterns of overstimulation enables proactive coping such as scheduling relaxing activities or alone time after a busy day. Identifying contributors to overstimulation can help individuals to develop strategies to improve the person-environment fit^26^. Qualitative studies suggest that combining avoidance, approach, and acceptance-based coping strategies can mitigate overstimulation^26,36^. To illustrate, when fatigued or in a negative mood, individuals may avoid sensory input by being alone or using noise-canceling headphones. While avoidance can offer short-term relief, it may worsen overstimulation at the long-term, aligning with the fear-avoidance model^49,50^. Graded exposure offers an alternative by gradually increasing sensory tolerance (e.g., listening to relaxing music instead of silence, or engaging in brief social interactions rather than full isolation), promoting habituation and reducing distress. Acceptance involves adjusting expectations and focusing on functionality rather than avoiding stimuli. It is central in therapies like Acceptance and Commitment Therapy and Mindfulness Based Interventions, which have been used to manage overstimulation in individuals with acquired brain injury^51^ and autism^52^. However, systematic research on coping strategies in the general population remains limited.

In the light of our results, HSPs may benefit more than non-HSPs from momentary pleasant visual and auditory stimuli (e.g., ambient lights, relaxing music) and from activities that enhance positive mood (e.g., nature walks). This aligns with research showing that HSPs feel more connected to nature^53,54^ and benefit more from virtual nature videos in terms of positive affect^55^. At the same time, increasing pleasant sensory input may not be universally beneficial. When the overall sensory load is high or when multiple competing stimuli are present (e.g., pleasant and unpleasant sounds occurring simultaneously), the introduction of an additional stimulus may fail to reduce overstimulation or may even amplify it^25^. Therefore, interventions should target overall sensory complexity and prioritize the reduction or regulation of multisensory inputs before simply adding a pleasant stimulus into an already overloaded environment.

Our results also indicate that HSPs were more affected by high levels of fatigue than non-HSPs, suggesting that improving sleep quality might be particularly important for preventing overstimulation in HSPs. Reducing overstimulation may be important for supporting mental health in HSPs^56^ as overstimulation can interfere with adaptive emotion regulation. Although heightened reactivity to everyday stimuli can be beneficial in pleasant environments, overstimulating environments may make it harder for HSPs to identify and regulate their emotions effectively^57^. Several studies show that HSPs are more likely to use maladaptive emotion regulation strategies^58–60^, and may experience reduced emotional awareness or alexithymia^61,62^, which further impairs adaptive emotion regulation^63,64^. Given that adaptive strategies (e.g., reappraisal) support wellbeing, while maladaptive strategies (e.g., suppression and avoidance) increase the risk for anxiety and depression ^[e.g., 65^, reducing overstimulation may be particularly important for promoting healthy emotion regulation and mental health in HSPs.

An important strength of this study is its novel focus on overstimulation in daily life within a general population sample, as prior research has mainly examined overstimulation in clinical groups (e.g., autism, stroke populations). Another strength is the robust ESM-design with high response rates (average response rates: 86.20%), enabling real-time assessment of momentary experiences. Previous studies have often relied on retrospective self-reports (e.g., recalling childhood experiences)^14,66^ or reporting the most positive and negative event of the day^40^ or week^39^. Additionally, earlier studies have primarily focused on negative experiences or the absence of negative events rather than the presence of positive ones^66^. Unlike experimental studies inducing positive experiences^10,16,17^, we assessed natural sensitivity to positive stimuli as they occurred in daily life.

Our study had also several limitations. First, the sample was skewed towards females, particularly in the HSP group, and included a higher proportion of older participants in the HSP group compared to the non-HSP group. Future studies should include more diverse samples to disentangle SPS effects from potential sex (e.g., hormonal cycles, social acceptance of sensitivity in women) or age-related influences. Second, although standard in personality research, the mere use of a self-report questionnaire to quantify SPS is a limitation. Additionally, predictors were analyzed separately due to high intercorrelations (e.g., mood and fatigue). Future studies should use larger samples and continuous SPS scores to detect more nuanced effects. Third, our analysis focused on momentary and one-directional associations with overstimulation as the outcome variable. Therefore, we cannot make any conclusions on the direction of effects. For example, it is likely that higher levels of overstimulation also influence fatigue, mood or sensory perceptions over time. Future research should explore bidirectional relationships between overstimulation and its predictors. Fourth, although we categorized high and low sense stimuli, we did not differentiate between specific stimuli within these categories (e.g., artificial light vs. sunlight) or considered participant’s control over these stimuli. Moreover, the present study assessed the pleasantness of different sensory modalities as they occurred naturally in participants’ environments but did not specifically examine multisensory combinations or cumulative sensory load (e.g., relaxing music occurring alongside background conversations or traffic noise). Therefore, our findings do not allow conclusions about whether adding a pleasant stimulus in a multisensory environment could offset, interact with, or potentially exacerbate overstimulation when other stimuli are present. Future studies should incorporate more detailed assessments of multisensory combinations, total sensory load, and perceived control over the sensory environment to better capture how complex sensory contexts influence overstimulation. Lastly, the present study did not assess broader-life context factors, such as occupational demands, caregiving responsibilities, or recent life events. Previous studies found that in some contexts (e.g., COVID-19, health care professionals, education professionals, and caregivers) individuals might be more prone to overstimulation than in other ones^67–69^. Incorporating these factors into future studies would help clarify how contextual demands interact with individual sensitivity to shape daily overstimulation.

To conclude, this study was the first to examine daily fluctuations in overstimulation and its associated factors in a general population sample with varying levels of SPS. Overstimulation peaked in the afternoon to early evening but did not fluctuate throughout the week. Higher levels of overstimulation occurred in the presence of others, with unpleasant sensory stimuli, and during periods of fatigue and negative mood. HSPs were not only more affected by negative internal and external triggers (i.e., fatigue, negative mood, unpleasant sensory stimuli) but also benefited more from momentary positive auditory and visual stimulations, low levels of fatigue, and a positive mood compared to non-HSPs. Raising awareness of these patterns may support individuals to develop coping strategies managing overstimulation, with potentially greater benefits for HSPs. Future research should investigate multisensory combinations, total sensory overload, perceived control, coping strategies to handle overstimulation, and bidirectional relationships between overstimulation and its triggers.

## Methods

### Participants and procedure

The study was approved by the university ethics committee of KU Leuven [G-2022-5629-R2(MAR)]. Healthy participants (i.e., without having any self-reported neurological, neurodevelopmental, or mental disorder) were drawn from a larger online questionnaire study (*N* = 848), recruited via social media channels targeting the general population and via the course participation in research for first year psychology students. Exclusion criteria were non-fluency in Dutch and self-reported neurological, neurodevelopmental, or mental disorders. The larger questionnaire study assessed SPS using the Sensory Processing Sensitivity Questionnaire^47^. Based on total SPSQ scores, participants were grouped into low (30% lowest scores on SPS), medium (40% middle SPS scores), and high sensitivity (30% highest SPS scores). This classification aligns with identified sensitivity levels using latent profile analyses across different samples and including different measures of SPS^10,70,71^. All participants from the larger study who left their contact details to be invited for follow-up studies were invited for the current study, independently of their SPS score. In total 160 adults accepted the invitation and signed a second informed consent form before the start of the current study. Since the sample was not equally distributed regarding participants’ SPS score (Supplementary Material E; Fig. E1), the sample was divided into a HSP and a non-HSP group. The latter was formed by combining the previously identified low and medium sensitivity groups. Participants completing less than 30% of ESM questionnaires (< 12 out of 35 observations, *n* = 21) were excluded^72^. A MCAR test^73^ suggested that data were missing completely at random (χ^2^ = 26412, *df* = 28297, *p* = 1). The final sample included 139 adults (*M*_age_ = 35.2; *SD*_age_= 12.6; range_age_ = 17–63; 84.2% female) with 51.8% (*n* = 72) classified into the HSP group, and 48.2% (*n* = 67) in the non-HSP group. The demographics of the total sample, as well as the HSP and non-HSP groups, are presented in Table 5.

Participants completed the ESM-questionnaires via the m-Path app^74^ on their smartphone five times a day for seven consecutive days. Prompts were sent at semi-random times within two-hour blocks between 7am and 9pm or between 8am and 10pm, based on participants’ self-identified morning or evening rhythms, to ensure they were likely awake. Questionnaires could be completed until the next prompt. As an incentive, participants had the opportunity to win a noise-cancelling headphone through lottery.

**Table 5:** Demographics of the total sample, the HSP group, and the non-HSP group.

	Total sample	HSP group	Non-HSP group	t χ²
*N*	139	72	67	
Age				
Mean	35.20	39.17	31.63	-3.61***#
Standard deviation	12.60	11.10	13.30	
Range	17–63	18–63	17–63	
Sex				
% female	84.20%	90.30%	77.60%	4.18*^
Nationality				
Belgium	92.10%	91.70%	92.50%	0.10^
The Netherlands	3.60%	7.00%	4.50%	0.40^
Germany	0.07%	1.40%	/	0.94^
Syria	1.40%	/	3.00%	1.08^
Highest educational level				
Primary school	1.50%	1.50%	1.60%	< 0.01^
Secondary school	34.60%	26.90%	42.90%	3.71^
Higher education (minimum a bachelor’s degree)	63.80%	71.60%	55.60%	3.00^

Sex was coded as 1 = female, 2 = male; *# = Independent* sample t-test (equal variances are not assumed); ^= Chi-Square test.

* *p* < .05; ** p < .01; *** *p* < .001.

### Measures

#### Sensory processing sensitivity

SPS was measured once, prior to starting on the ESM, with the Sensory Processing Sensitivity Questionnaire (SPSQ)^47^. All 43 items were rated on a 7-point Likert scale (1 = *not at all*, 4 = *moderately*, 7 = *extremely*). The total mean score of the SPSQ, with an excellent internal consistency (α = 0.95), was used.

#### Experience sampling methodology

The ESM questionnaire (Supplementary Material F) included: one item assessing current levels of overstimulation, 15 items evaluating the pleasantness of stimuli in their current environment, two items assessing current levels of fatigue, 19 items measuring current mood, and five items assessing the type of current environment. Descriptive statistics and response rates are presented in Table A1 (Supplementary Material A).

##### External triggers

*(a) Sensory stimuli.* Participants rated the pleasantness of various stimuli (e.g., lights, sounds, smells, tastes), if applicable, on a scale from 1 (*not at all*) to 7 (*extremely*). In addition, participants rated their level of stimulation on a scale from − 3 (*very understimulated*) to + 3 (*very overstimulated*), later converted to a 1 to 7 scale with higher scores indicating greater overstimulation. Based on theory^30^, exploratory and confirmatory factor analyses (Supplementary Material G, Table G1), stimuli were grouped into high (i.e., lights, sounds, bright colors, moving scenes, music, multiple stimuli simultaneously; α = 0.86) and low (i.e., touches, smells, tastes, temperature; α = 0.72) senses. Items were developed by the authors of the current study based on a questionnaire study assessing multisensory sensitivity^29^ as no prior ESM study has examined this construct. *(b) Type of environment.* Participants selected their current location (e.g., at home, with family or friends, at work). They reported the presence of others, specifying whether they were alone or with others and with whom (e.g., friends, colleague, stranger, partner). Two dummy variables for the type of environment were created: location (private (0) versus public (including at work or school; 1) and social context (alone (0) versus with others (1)).

##### Mood

Participants rated their current mood on a scale from 1 (*not at all*) to 7 (*extremely*), assessing both positive (happy, satisfied, relaxed, at ease, relieved, energetic, and good) and negative moods (irritated, anxious, uncertain, lonely, guilty, suspicious, sad, restless, gloomy, lethargic, agitated, and stressed). These items are selected from the ESM item repository (https://esmitemrepositoryinfo.com/) and are based on the Positive and Negative Affect Scale (PANAS)^75^. Due to high intercorrelations, a composite mood score (α = 0.96) was calculated by reversing negative mood items and averaging with positive mood items; higher scores indicate a more positive mood.

##### Fatigue

Fatigue was measured using two items from the ESM item repository: ‘How physically tired are you?’ and ‘How mentally tired are you?’. Both items were answered on a scale from 1 (*not at all*) to 7 (*extremely*). The mean of both items was calculated as a measure of fatigue (α = 0.87).

### Data analysis

First, we examined descriptive statistics, including within-person means, between-person means, standard deviations, Pearson bivariate correlations, frequencies, and response rates. Second, multilevel models were estimated using restricted maximum likelihood (REML). Multilevel models are the recommended method to analyze ESM data as this type of data adhere to a multilevel structure including repeated measurements nested within days and individuals^42,72^. Minimum sample size estimations were based on guidelines for multilevel modeling^76^ recommending at least 50 clusters (participants) per group with at least 30 observations each, since we did not have any estimates on effect sizes for the studied variables. Momentary observations as predictor variables were nested within individuals, random intercepts and random slopes were estimated. Overstimulation was the outcome variable. In each model the main effect of SPS (HSP versus non-HSP; between-person) as well as the interaction effects of SPS with the predictors were included. Per type of additional time-varying momentary (within-person) predictor, a separate model was estimated: (1) time of the day (i.e., prompt) and day of the week (i.e., weekday ranging from Monday to Sunday), (2) type of the environment (i.e., location and social context), (3) pleasantness of stimuli in the current environment, (4) current levels of fatigue, and (5) current levels of mood. In models 2 to 5, the predictors were within-person mean centered by distracting the subject-level means from the original variable. Based on visual exploration of the raw data (Fig. 1), prompt was included as normal and squared coefficient. Third, we reran the same multilevel models (models 2–5) with type of the environment, pleasantness of stimuli, fatigue, and mood as lagged to the previous measurement moment (t-1). For all multilevel models, we corrected for multiple testing to avoid false positive findings by using False Discovery Rate^77^. Significant interactions using simple slopes, including Regions of Significance (RoS) with respect to X (i.e., at which values of the predictor the effect of regression is statistically significant) and the Proportion of the Interaction^23^ (PoI; the proportion of the total interaction that is represented by the left and the right sides of the crossover point) were plotted. We considered PoI values between 0.20 and 0.80 as evidence for Differential Susceptibility, with values close to 0 as evidence for Diathesis-stress and close to 1 as evidence for Vantage Sensitivity, based on the most recent guidelines^78^. As more older and female participants were in the HSP compared to the non-HSP group (Supplementary Material H, Fig. H1), we controlled for age and sex effects as an exploratory analysis (Supplementary Material D).

### Transparency and openness

We report how we determined our sample size, all data exclusions, manipulations, measures, procedures, and results transparently, and we follow JARS^79^. All analyses were run in SPSS Version 28^80^ and R version 4.3.2.^81^ using packages *esmpack*^82^, *nlme*^83^, *lavaan*^84^, and *ggplot2*^85^. This study was not preregistered on OSF before data collection started due to tight project timelines. However, all hypotheses, study design, and planned analyses were outlined in detail in a grant proposal and approved before data collection started.

### Ethical approval

The study was approved by the university ethics committee of KU Leuven [G-2022-5629-R2(MAR)]. All procedures performed in studies involving human participants were in accordance with the ethical standards of the institutional committee and with the 1964 Helsinki declaration and its later amendments or comparable ethical standards. This article does not contain any studies with animals performed by any of the authors.

### Informed consent

Informed consent was obtained from all individual participants included in the study.

## Supplementary Information

Below is the link to the electronic supplementary material.

Supplementary Material 1

## Data Availability

A link to the anonymized data and analysis code is made available on Open Science Framework (OSF): [https://osf.io/598fh/?view_only=00c6acc7361249fbbf24f042985752a7] .
