# Supplementary material for: Sensory processing sensitivity and overstimulation in daily life: an experience sampling method study
Source: Sci Rep. 2025 Dec 18;16:2015. doi: 10.1038/s41598-025-31629-3 (PMC12808265; doi:10.1038/s41598-025-31629-3)
Supplement: Supplementary file 1 — Supplementary Material 1 [file 41598_2025_31629_MOESM1_ESM.docx]

**Supplementary Material A**

**Descriptive Statistics and Response Rates**

**Table A1**

*Means, Standard Deviations, Frequencies, and Response Rates for Each Variable*

| **Variable** | ***M* (*SD*)**  **Total sample** | ***M* (*SD*)**  ***Per group*** | | ***M (SD)* response rates (%)** | **Range**  **Response rates (%)** |
| --- | --- | --- | --- | --- | --- |
|  | ***N* = 139** | **HSP (*n* = 72)** | **Non-HSP (*n* = 67)** |  |  |
| General response rate (%) | 86.20 (16.50) | 84.88 (17.18) | 87.56 (15.69) | 86.20 (16.50) | 34.40- 100 |
| Overstimulation | 4.34 (0.54) | 4.43 (0.52) | 4.24 (0.55) | 86.20 (16.50) | 34.40- 100 |
| SPS | 5.04 (0.86) | 5.73 (0.33) | 4.26 (0.53) | 97.20 (14.90) | 0-100 |
| Pleasantness Higher Senses | 4.02 (0.68) | 3.72 (0.61) | 4.34 (0.60) | 84.40 (17.70) | 28.60-100 |
| Pleasantness Lower Senses | 4.61 (0.56) | 4.49 (0.56) | 4.73 (0.55) | 84.30 (18.20) | 20-100 |
| Mood | 4.51 (0.79) | 4.27 (0.79) | 4.77 (0.71) | 84.40 (17.70) | 28.60-100 |
| Fatigue | 4.24 (1.09) | 4.64 (0.99) | 3.80 (1.01) | 84.20 (18.20) | 17.10-100 |
| Alone versus with others (whom) | 49.50% with others | 49.40% with others | 49.60% with others | 66.10 (21.70) | 8.60-97.10 |
| Private versus public (where) | 30.20% in public environments | 27% in public environments | 34.20% in public environments | 77.10 (19) | 17.10-100 |

*Note.* SPS = Sensory Processing Sensitivity, categorized as HSP versus non-HSP. For all time-variant continuous variables the within-person means were calculated, for SPS a between-person mean was calculated. Mood was coded with scores higher scores indicating positive mood; Fatigue entails both physical and mental fatigue. For the type of the environment (alone versus with others and private versus public) frequencies were reported.

Sensitivity analysis: We tested whether the average response rates were influenced based on participant’s average mood and fatigue levels. At the between-person level, response rates were not associated with participants’ average fatigue levels (*r* = .08, *p* = 0.34) but were weakly with participants’ average mood levels (*r* = -.21, *p* = 0.02).

**Supplementary Material B**

**Pearson Correlations**

**Table B1**

*Pearson Correlations Between the Continuous Variables Included in the Analyses*

| Variable | 1. | 2. | 3. | 4. | 5. | 6. |
| --- | --- | --- | --- | --- | --- | --- |
| 1.Overstimulation | - |  |  |  |  |  |
| 2.SPS | .11*** | - |  |  |  |  |
| 3.Pleasantness Higher Senses | -.19*** | -.25*** | - |  |  |  |
| 4.Pleasantness Lower Senses | -.10*** | -.13** | .45*** | - |  |  |
| 5.Mood | -.19*** | -.30*** | .39*** | .35*** | - |  |
| 6.Fatigue | .13*** | .24*** | -.23*** | -.24*** | -.56*** | - |
| *Note.* SPS = Sensory Processing Sensitivity; Mood was coded with higher scores indicating positive mood; Fatigue entails both physical and mental fatigue; ***p* < .01; *** *p* < .001. | | | | | | |

**Supplementary Material C**

**Significant Interactions with Lagged Predictors**

**Figure C1**


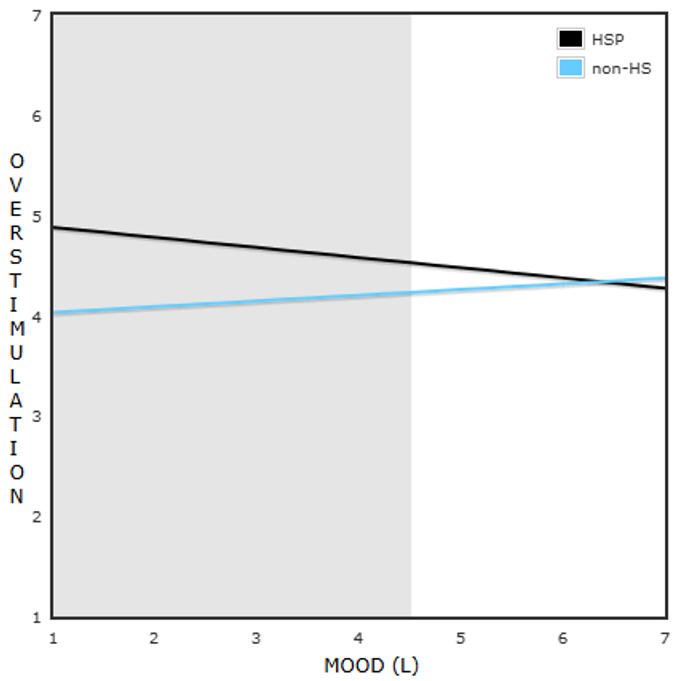

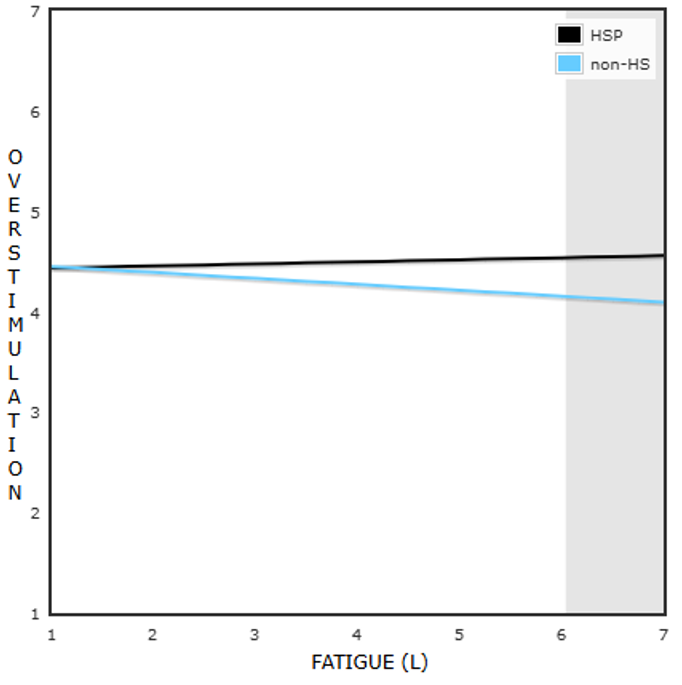
*
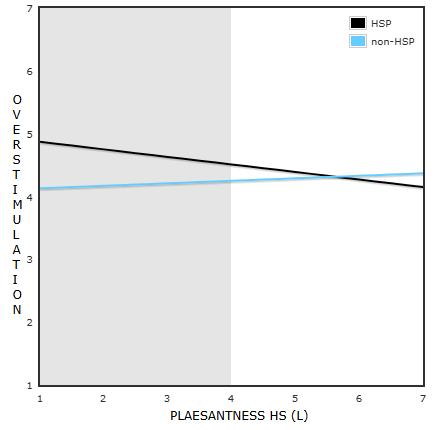
Overstimulation as Predicted by the Interactions of SPS and Pleasantness of High Sense Stimuli (Top Left), Fatigue (Top Right), and Mood (Bottom) as Reported at the Previous Prompt (Lagged Variables)*

*Note.* The grey areas represent the Regions of Significance with respect to X (i.e., Pleasantness high senses, Fatigue, or Mood). HS= high sense stimuli; L = lagged.

**Supplementary Material D**

**Controlling for Age and Sex**

**Table D1**

*Multilevel Random Intercept and Slope Model Results Predicting Fluctuations in Overstimulation Including Age and Sex as Covariates (Model 1)*

| **Model 1** | **Β (SE)** | ***p* -value** |
| --- | --- | --- |
| Intercept | 4.08*** (0.15) | <.001 |
| Age | 0.00 (0.01) | 0.48 |
| Sex | -0.22 (0.12) | 0.08 |
| Time of the day | 0.45*** (0.08) | <.001 |
| Time of the day² | -0.07*** (0.01) | <.001 |
| Weekday | -0.07 (0.05) | 0.17 |
| SPS | 0.15 (0.13) | 0.28 |
| Time of the day*SPS | 0.04 (0.12) | 0.72 |
| Time of the day²*SPS | -0.01 (0.02) | 0.64 |
| Weekday *SPS | -0.01 (0.08) | 0.38 |
| ICC | .25 |  |
| *R*² (fixed effects) | .06 |  |
| *R*² (total) | .30 |  |

*Note.* SPS = Sensory Processing Sensitivity, categorized as HSP versus non-HSP; Time of the day² is the quadratic term of time of the day. ICC= Intraclass correlation coefficient. *R²* = proportion of variance explained by the fixed effects and/or fixed and random effects. Sex was coded with 1 as Female and 2 as Male.

*** *p* < .001.

**Table D2**

*Multilevel Random Intercept and Slope Model Results Predicting Overstimulation by SPS, the Pleasantness of Stimuli in the Current Environment, the Type of Environment, Mood and Fatigue, and its interactions, Including Momentary and Lagged Variables (Model 2-5), Including Age and Sex as Covariates*

|  | **Within-person centered predictors (momentary observations)** | | **Time-lagged predictors (previous observation)** | |
| --- | --- | --- | --- | --- |
|  | **Β (SE)** | ***p* -value** | **Β (SE)** | ***p* -value** |
| **Model 2: Pleasantness of stimuli** |  |  |  |  |
| Intercept | 4.08*** (0.12) | <.001 | 4.06*** (0.23) | <.001 |
| Age | 0.01 (0.00) | 0.575 | 0.00 (0.00) | 0.579 |
| Sex | -0.17 (0.11) | 0.110 | -0.23 (0.13) | 0.080 |
| Pleasantness high senses | -0.11* (0.05) | 0.022 | 0.05 (0.03) | 0.190 |
| Pleasantness low senses | -0.10** (0.04) | 0.007 | 0.01 (0.04) | 0.880 |
| SPS | 0.13 (0.09) | 0.15 | 0.86** (0.27) | 0.002 |
| Pleasantness high senses * SPS | -0.23*** (0.07) | <.001 | -0.17** (0.05) | 0.001 |
| Pleasantness low senses * SPS | 0.02 (0.05) | 0.683 | -0.01 (0.06) | 0.920 |
| ICC | .25 |  | .20 |  |
| *R*² (fixed effects) | .06 |  | .02 |  |
| *R*² (total) | .30 |  | .21 |  |
| **Model 3: Type of environment** |  |  |  |  |
| Intercept | 4.12*** (0.22) | <.001 | 4.23*** (0.14) | <.001 |
| Age | 0.00 (0.00) | 0.586 | 0.00 (0.00) | 0.593 |
| Sex | -0.15 (0.12) | 0.200 | -0.22 (0.13) | 0.086 |
| Alone versus with others | 0.43** (0.14) | 0.002 | 0.15*(0.07) | 0.023 |
| Private versus public | 0.43* (0.20) | 0.030 | 0.05 (0.07) | 0.437 |
| SPS | 0.18 (0.09) | 0.060 | 0.10 (0.11) | 0.367 |
| Alone versus with others * SPS | -0.17 (0.09) | 0.060 | -0.12 (0.10) | 0.239 |
| Private versus public * SPS | 0.05 (0.13) | 0.730 | 0.14 (0.11) | 0.182 |
| ICC | .23 |  | .39 |  |
| *R*² (fixed effects) | .06 |  | .01 |  |
| *R*² (total) | .27 |  | .39 |  |
| **Model 4: Fatigue** |  |  |  |  |
| Intercept | 4.12*** (0.11) | <.001 | 4.53*** (0.15) | <.001 |
| Age | 0.00 (0.00) | 0.143 | 0.00 (0.00) | 0.603 |
| Sex | -0.14 (0.11) | 0.210 | -0.28* (0.11) | 0.017 |
| Fatigue | 0.02 (0.03) | 0.495 | -0.06* (0.03) | 0.01 |
| SPS | 0.14 (0.09) | 0.134 | -0.15 (0.16) | 0.333 |
| Fatigue *SPS | 0.13** (0.05) | 0.003 | 0.08* (0.04) | 0.023 |
| ICC | .23 |  | .20 |  |
| *R*² (fixed effects) | .03 |  | .02 |  |
| *R*² (total) | .25 |  | .21 |  |
| **Model 5: Mood** |  |  |  |  |
| Intercept | 4.15*** (0.12) | <.001 | 3.89*** (0.28) | <.001 |
| Age | 0.00 (0.00) | 0.341 | 0.00 (0.00) | 0.289 |
| Sex | -0.07 (0.11) | 0.558 | -0.26* (0.12) | 0.035 |
| Mood | -0.12 (0.07) | 0.055 | 0.06 (0.04) | 0.178 |
| SPS | 0.16(0.09) | 0.094 | 0.98** (0.35) | 0.006 |
| Mood*SPS | -0.27** (0.09) | 0.002 | -0.16** (0.06) | 0.006 |
| ICC | .26 |  | .21 |  |
| *R*² (fixed effects) | .05 |  | .02 |  |
| *R*² (total) | .30 |  | .22 |  |

*Note.* SPS = Sensory Processing Sensitivity, categorized as HSP versus non-HSP; Alone was coded as 0 and with others as 1. Private was coded as 0 and in public places (including the work or school environment) as 1. ICC= Intraclass correlation coefficient. *R²* = proportion of variance explained by the fixed effects and/or fixed and random effects. Sex was coded with 1 as Female and 2 as Male.

**p* < .05; ***p* < .01; *** *p* < .001

**Supplementary Material E**

**The Distribution of SPS scores**

**Figure E1**

*The Distribution of SPS scores among the Final Sample, as a Continuous Score (a) and a Categorical Score (b)*

**a**

*
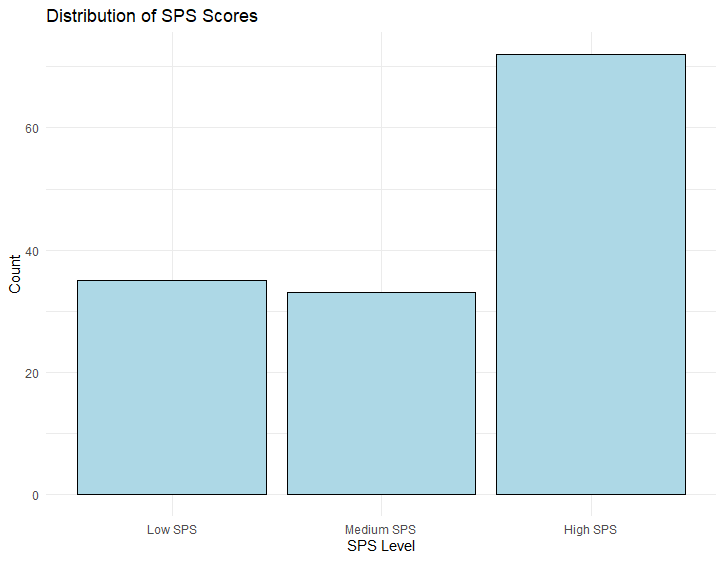
*
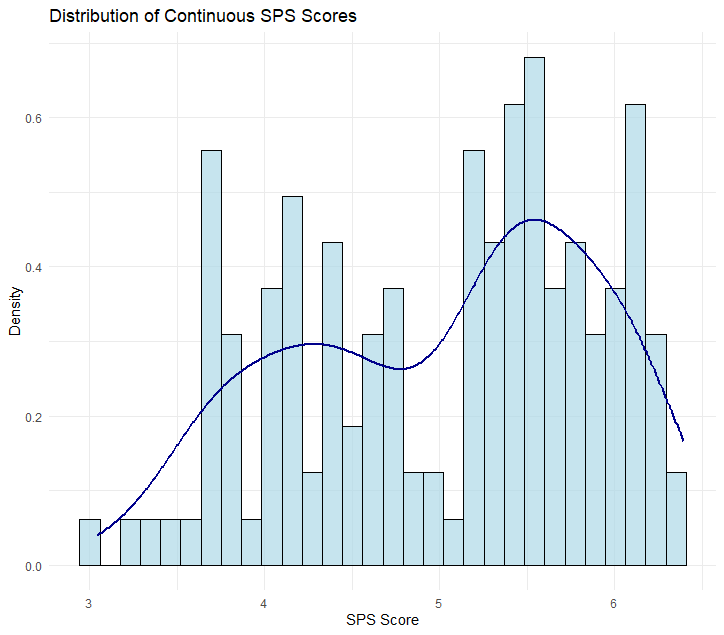


**b**

**Supplementary Material F**

**Experience Sample Method questionnaire**

Stimulation:

How much are you currently under- or overstimulated (-3 to +3)

Pleasantness sensory stimuli and stimulation

- - - How unpleasant to pleasant would you rate below stimuli, if present in your current environment: (scale: 1-7)
      - Lights
      - Sounds
      - Bright colors
      - Being touched (by others or by clothes)
      - Tastes
      - Smells
      - Moving objects (passing cars)
      - Art
      - Music
      - Temperature
      - Multiple stimuli at the same time
      - Other:….

Fatigue

- - - How physically tired are you? (1-7)
    - How mentally tired are you? (1-7)

Mood

- - - How do you currently feel (mood): scale: 1-7
      - Happy (pos)
      - Irritated (neg)
      - Anxious (neg)
      - Satisfied (pos)
      - Uncertain (neg)
      - Lonely (neg)
      - Guilty (neg)
      - Relaxed (pos)
      - At ease (pos)
      - Suspicious (neg)
      - Sad (neg)
      - Restless (neg)
      - Gloomy (neg)
      - Relieved (pos)
      - Lethargic (neg)
      - Agitated (neg)
      - Stressed (neg)
      - Energetic (pos)
      - Good (pos)

Environment

- - - **Where are you at the moment?**
      - At home
      - With family or friends
      - At work or at school
      - Health care
      - Public activity (cinema, concert, …)
      - On the way (on the street, in the train, on the bike)
      - In nature
      - In the supermarket/bakery
      - In a bar or restaurant
      - In the sport club
      - Somewhere else:….
    - **At the moment I am with:**
      - Nobody
      - Friend(s)
      - Colleague, classmate
      - My partner
      - Parent(s)
      - Brother(s)/sister(s)
      - Family (e.g., aunt, uncle, grandparents)
      - Acquaintance(s)
      - A health care provider (e.g., doctor, psychologist, physiotherapist)
      - Teacher
      - Roomie(s)
      - Unknown other(s)
      - Other: …..

**Supplementary Material G**

**Exploratory and Confirmatory Factor Analyses Pleasantness of Stimuli**

Based on theory (Köster, 2003) we checked whether we could reduce the number of dimensions measuring the pleasantness of stimuli across the different modalities by running an Exploratory Factor Analysis (EFA) using principal component analyses and varimax rotation. A multilevel CFA was run in R (package Lavaan) with observations nested within persons, with Full Information Maximum Likelihood (FIML) estimation to deal with missing data and using robust maximum likelihood (MLR) estimation to control for non-normality, to check the model fit indices of the suggested factor structure of the EFA. An acceptable fit is obtained when the Comparative Fit Index (CFI) is .90 or above, and the Mean Root Square Error of Approximation (RMSEA) and Standardized Root Mean Squared Residual (SRMR) are below 1 (Kline, 2016). Results of the EFA showed evidence for a two-factor solution. Factor 1 included lights, sounds, bright colors, moving scenes, music, and multiple stimuli at the same time, further referred to as high sense stimuli. Factor 2 included touches, smells, tastes, and temperature, further referred to as low sense stimuli. Multilevel CFA showed an acceptable model fit for the two-factor solution (CFI = .90, RMSEA = .06, SRMR = .06). The factor loadings are reported in Table G1.

**Table G1**

*Standardized Factor Loadings of the Multilevel CFA Regarding Pleasantness of Stimuli Across Modalities*

| **Pleasantness of Stimuli** | **Pleasantness of High Sense (λ1)** | **Pleasantness of Low Sense**  **(λ2)** |
| --- | --- | --- |
| Lights | 0.75*** |  |
| Sounds | 0.71*** |  |
| Bright colors | 0.74*** |  |
| Moving scenes | 0.63*** |  |
| Music | 0.43*** |  |
| Multiple stimuli | 0.52*** |  |
| Touches |  | 0.55*** |
| Smells |  | 0.48*** |
| Tastes |  | 0.40*** |
| Temperature |  | 0.52*** |
| *Note.* **Λ** = standardized factor loading; *** *p* < .001. | | |

Reference:

Köster, E. P. (2003). The psychology of food choice: Some often encountered fallacies. *The Sixth Sense - 6th Sensometrics Meeting*, *14*(5), 359–373. https://doi.org/10.1016/S0950-3293(03)00017-X

**Supplementary Material H**

**The Distribution of Age and Sex According to the SPS Scores**

**Figure H1**

*The Distribution of Age (a) and Sex (b) According to the SPS Scores*


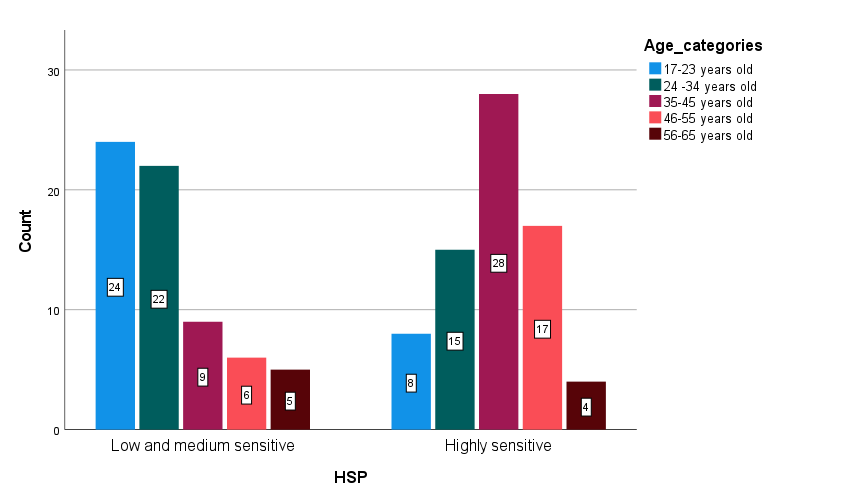


**a**


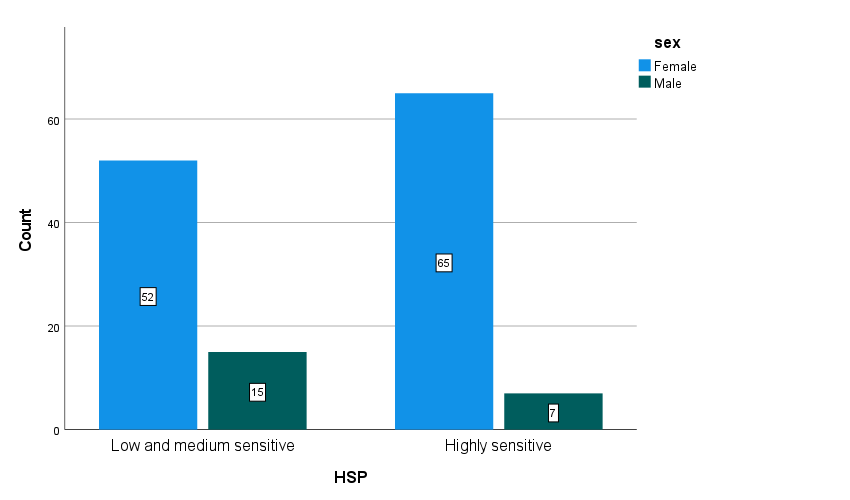


**b)**
